# The severity of dental caries in adults aged 35 to 44 years residing in the metropolitan area of a large city in Brazil: a cross-sectional study

**DOI:** 10.1186/1472-6831-12-25

**Published:** 2012-07-31

**Authors:** Simone M Costa, Mara Vasconcelos, João Paulo A Haddad, Mauro Henrique NG Abreu

**Affiliations:** 1 Department of Dentistry, Universidade Estadual de Montes Claros, Montes Claros, Brazil; 2 Department of Community and Preventive Dentistry, Universidade Federal de Minas Gerais, Av. Antônio Carlos, 6627, ZIP Code 31270901, Belo Horizonte, Minas Gerais, Brazil; 3 Department of Veterinary Preventive Medicine, Universidade Federal de Minas Gerais, Belo Horizonte, Brazil

**Keywords:** Socioeconomic factors, Oral health, Epidemiology

## Abstract

**Background:**

In recent decades, studies in the field of public health have increasingly focused on social determinants that affect the health-illness process. The epidemiological perspective considers oral health to be a reflection of socioeconomic and environmental aspects, and it is particularly influenced by the social context. The aim of the present study was to assess the association between the severity of dental caries among adults aged 35 to 44 years and characteristics on the different levels at which the determinants of caries operate (individual, social structure and social context).

**Methods:**

A home-based, cross-sectional field study was carried out involving a sample of 1,150 adults (35 to 44 years of age) residing in metropolitan Belo Horizonte, Brazil. The DMFT (decayed, missing, filled tooth) index (≥14) was used to determine the severity of dental caries. Bivariate and multivariate analyses were carried out using the Poisson regression model with the level of significance set at 5% (p < 0.05) and 95% confidence intervals.

**Results:**

The majority of the participants (68.5%) had high caries severity. The rate of high-severity caries in the group between 40 and 44 years of age was 1.15-fold (CI: 1.04-1.26) greater than that among those aged 35 to 39 years. A greater prevalence of high caries severity was found among those who frequently visited the dentist (PR = 1.18; CI: 1.07-1.30), those with a lower income (PR = 1.11; CI: 1.01-1.23), those who reported that their neighborhood did not come together in the previous year to petition political leaders for benefits (PR = 1.16; CI: 1.05-1.28) and those who are unable to make decisions (without empowerment) (PR = 1.12; CI: 1.01-1.24).

**Conclusions:**

The present study revealed high dental caries severity in adults, which was associated with individual characteristics, health-related behavior and social structure and contextual variables. These findings underscore the importance of considering social determinants involved in the health-illness process when carrying out epidemiological studies on dental caries.

## Background

In recent decades, studies in the field of public health have increasingly focused on social determinants in the health and illness process [1]. Considering oral health as a reflection of socioeconomic and environmental characteristics [2], epidemiological studies draw greater attention to the effect of the social context on health [3]. Investigations carried out in Canada [4] and the United Kingdom [5] demonstrate that the social context exerts a considerable influence over the oral health status of adults in areas in which there is a lack of access to material resources and community participation.

Social epidemiology has made great advances over the past three decades at a time when health inequalities have widened across countries. There are many theoretical frameworks that address the social context and its interaction with biological and psychological factors. Psychosocial factors, i.e. social isolation, explain why particular social groups are disproportionately affected by different diseases. Social production of disease/political economy of health advocated the materialist analysis of health and introduced the “upstream-downstream” metaphors. Ecosocial and other multi-level theories seek to integrate social and biological factors in a dynamic, historical and ecological perspective [6]. Recently, it was reinforced that social class or socioeconomic position, is not only a striking predictor of disease occurrence, but the associations reflects causal connections [7].

In this sense, theories have emerged addressing the social context and its interaction with both biological and psychological factors [3-8]. Studies have also stressed the importance of socioeconomic factors in the health-illness process [9]. The position that a social group occupies in society can lead to a greater or lesser risk of disease as well as greater or lesser access to healthcare services [3-10].

Studies on social inequity and health have explored the interactions between context and individual variables [10,11]. The behavior of an individual (such as lifestyle aspects) and income have been associated with various diseases [10,11]. Income is considered a socioeconomic measure related to material conditions and a differentiating factor regarding access or non-access to health services. Income affects eating patterns, housing, knowledge and access to health care, all of them directly affect either exposure to risk or protection from disease [10]. Education is also considered an important component of socioeconomic status that contributes to health differences [12]. Moreover, psychological wellbeing, a lack of stress and access to social networks are factors that have been attributed to health maintenance. Thus, biological factors are influenced by economic, social and psychological factors in the development of chronic diseases [11], such as dental caries [2,3].

Social and economic inequities and their impact on health on both an individual and a population basis are important public health issues [10]. However, there are few epidemiological studies that consider the different levels on which the determinants of dental caries operate [3], and this has hindered the organization and implementation of adequate health promotion strategies. Thus, the main contribution of the present population-based epidemiological study is the description of the severity of dental caries among adults residing in urban areas, considering the different levels on which the possible determinants of caries operate.

A recent epidemiological survey on oral health involving adults in Brazil (Oral Health Brazil 2010) did not employ valid sampling methods for metropolitan regions. Thus, to the best of our knowledge, the present study is the first epidemiological survey on oral health regarding dental caries among adults aged 35 to 44 years to be carried out in a metropolitan region of a major city in Brazil; no previous similar epidemiological study was found in the PubMed database as of January 2012 based on searches using the descriptors “epidemiology”, “caries” and “Brazil” and considering an age range of 19 and 44 years.

The hypothesis of the present study is that a greater severity of dental caries among adults is influenced by the demographic characteristics of individuals and their health-related behavior as well as variables related to the social structure and context in which individuals reside. The aim was to assess the association between caries severity in adults aged 35 to 44 years and the characteristics of this population with respect to the different levels at which the determinants of caries operate (individual, social structure and social context levels).

## Methods

This study is part of a broader-scoped study titled **“**Oral Health among Adults in Metropolitan Belo Horizonte (urban areas): Objective and Subjective Aspects”, and it received approval from the Human Research Ethics Committee of the *Universidade Federal de Minas Gerais* (Brazil) under process number ETIC 096/09.

An analytical cross-sectional study was carried out involving data collection at the places of residence of the participants. The study population consisted of adults aged 35 to 44 years residing in urban areas of the municipalities surrounding Belo Horizonte, which is the capital of the state of Minas Gerais, Brazil. This latter was our inclusion criteria. Metropolitan Belo Horizonte has 33 municipalities and is the third largest metropolis in the country. The region is the political, financial, commercial, educational and cultural center of Minas Gerais, accounting for approximately 40% of the economy and 25% of the population of the state.

The adult population of metropolitan Belo Horizonte aged 30 to 49 years is estimated to be 785,439 individuals [13]. Based on this figure, the sample size was calculated using the prevalence of dental pain reported by adults as the main reason for seeking dental care (41.21%) [14]. The level of confidence was 95% (1- α), and the level of precision (d) was 10%. Dental pain was used because it is considered an important common outcome among individuals with dental caries, and it provided a larger sample size (575 individuals) compared to others outcomes. Due to the conglomerate sampling, *design effect (deff)* = 2 [15] was used to compensate for the loss of variability among the participants, leading to a sample of 1150 individuals. The sample was selected through probabilistic conglomerate sampling by stages, municipalities, districts, blocks and domiciles [15,16].

The 33 municipalities surrounding Belo Horizonte were first grouped according to population quartiles. The homogeneity among the municipalities in each quartile was then analyzed using municipal social indicators from the living conditions index (LCI-habitation and LCI-education). All municipalities within the same quartile were classified within a single evaluation category [17]. Thus, two municipalities from each stratum of number of inhabitants were randomly selected by lots, totaling eight municipalities to compose the sample. To determine whether the eight municipalities sampled represented the total number of 33 municipalities, tests were performed for the comparison of mean values between the sampled (n = 8) and non-sampled (n = 25) municipalities. The Mann–Whitney test was used to compare the means of the Human Development Index, and the Student’s t-test was used to compare the mean values of the LCI-habitation, LCI-education, LCI-income, LCI-healthcare services and synthesized LCI. No statistically significant differences were found between the mean values of the sample and the non-sampled groups regarding any of the social or economic indicators (p > 0.05).

The distribution of adults to make up the sample was proportional to the total number of adults aged 35 to 44 years in each of the eight municipalities selected. Using a map of each municipality, the districts, blocks and domiciles were randomly selected by lots to reach adults within the age range of interest.

The criteria of the World Health Organization regarding the determination of the decayed, missing and filled tooth (DMFT) index were used for the evaluation of dental caries. The exams were performed by five dentists, who were previously calibrated [18]. The reproducibility of the examiners in recording dental caries, measured by the inter-examiner and intra-examiner Kappa values, ranged from 0.81 to 0.92 and 0.80 to 1.00, respectively.

**Figure 1:**
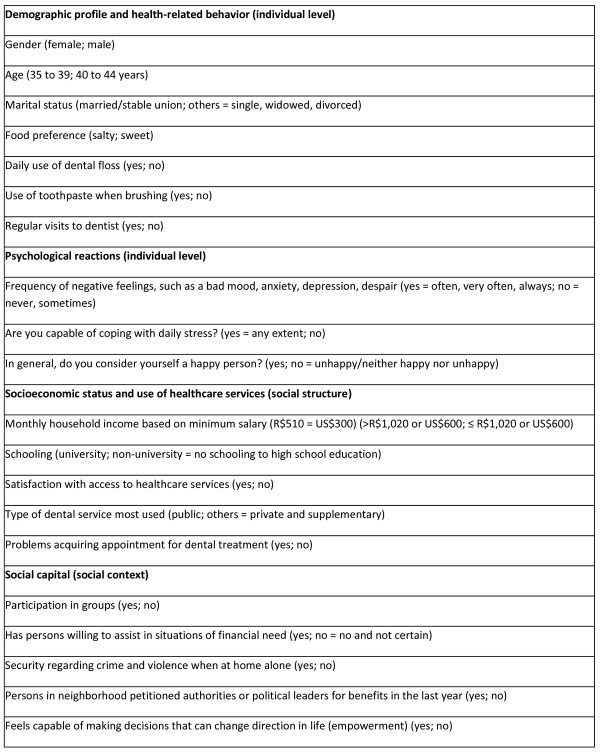
Independent variables employed in the bivariate and multivariate analyses.

For the definition of the severity of dental caries, DMFT ≥ 14 was considered high severity, and DMFT <14 was considered low severity [19]. The caries severity was tested for associations with dichotomized independent variables (Figure 1) based on the explanation structure of caries in the population [3]. This model has a network of determinant factors that are interlinked with the social structure and social context at the individual and biological levels. The hypothesis is that social structure in terms of health policies, political, and economic situations may affect the social context in different arenas and directly individual material resources. So, these conditions may affect behaviors and, finally, dental caries [3]. This theoretical framework [3] has defined the choice of indicators and instruments [14,20-23] used for each of its dimensions. These indicators are validated in Brazil [14,20-23]. The social structure comprises culture, health policies, the distribution and organization of healthcare services, the use of these services, economic status and schooling. The social context involves the nature of the surrounding neighborhood, local organization and the accessibility of healthcare services, family services and work services. The individual level incorporates psychological factors, health-related behaviors and material factors. The three psychological variables were chosen from three instruments [21-23]. The choice of variables that comprised the psychological level is because of stress, anxiety, depression and feelings of unhappiness function as endogenous biological factors possibly associated with the outcome of disease [6]. Moreover, psychological reactions have been included in the framework, because there is a growing recognition of the psychological aspects and relations in social life and health [3]. 

The biological level entails the ecology of caries [3]. Variables for the measurement of social capital were used in the social context category. The selection of the indicators of each one of the dimensions of our theoretical framework [3] was based on previous literature of dental caries. This model was chosen considering the eco-social theory [6] that was incorporated to epidemiology of dental caries [3].

A questionnaire based on the published literature was designed for the collection of the independent variables [14,20-22]. Although some questions were not formally validated, this questionnaire was tested in a pilot study to determine the understanding of adults. Moreover, the test-retest method was employed to assess the responses of 25 adults on two separate occasions. Concordance between the responses on the two separate administrations of the questionnaire was determined, and a high degree of reproducibility was demonstrated (kappa > 0.60). A questionnaire is more reliable when it is possible to reproduce the same responses on different occasions [24]. All variables were dichotomized.

The statistical analysis involved the use of the sampling weight to compensate for unequal probabilities among the elements of the sample [25]. Considering the conglomerate sampling design, natural expansion factors were used, which are differentiated weights for the elements of the sample, to compensate for unequal selection probabilities. This method is important because not taking the conglomerate sampling into account could produce errors in the mean values and respective variances in traditional statistical analysis, subsequently leading to incorrect results, hypothesis tests and conclusions [26]. The sample weight considered the total of the census sectors and blocks in each of the selected municipalities, the total number of individuals living in these residential areas and the number of individuals examined per census sector and block.

We performed a multi-level analysis to evaluate the influence of ecological variables, i.e. Human Development Index [data not shown] on the dental caries experience, using the program HLM 8.0. It was found that the dental caries experience was not different between the eight cities surveyed (p = 0.133). Thus, it was not necessary to evaluate the influence of ecological variables on dental caries.

Thus, the methodological approach analysis followed the one proposed by Victora *et al.*[27]. In this type of analysis is possible to characterize proximal and distal individual variables to the outcome and allows the selection of those most strongly associated with it.

**Figure 2:**
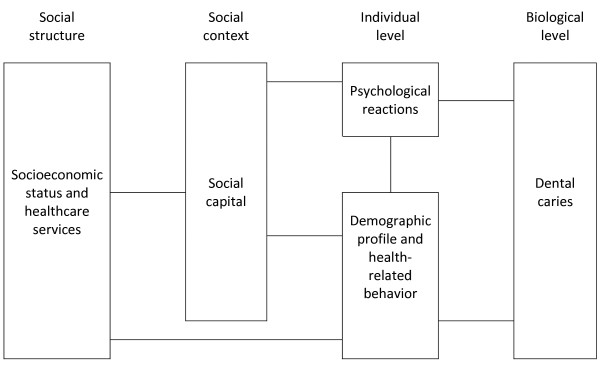
** An approach to a framework for explaining caries in populations**[3]

The measure of association used was the prevalence ratios with respective 95% confidence interval, estimated using Poisson regression with robust variance and Wald test. In a first step, bivariate analyzes were performed to set up the description of the characteristics of the study population and production of crude prevalence ratios (with corresponding 95% confidence intervals) for associations between covariates and dental caries. All variables that achieved a p-value ≤ 0.20 were incorporated into the multivariate model. Then, multivariate analyses were performed, considering the blocks of variables based on the dental caries model [3] and the hierarchical analysis adopted [27]. Initially, the model was shot on the set of variables of the hierarchical level more distal (social structure), followed by the inclusion of other blocks of variables according to the sequence described in Figure 2. Those that achieved a p-value ≤ 0.05 were considered significant [28]. The statistical analysis was performed using the Statistical Package for the Social Sciences (SPSS 18.0). 

## Results

A total of 1,150 adults aged 35 to 44 years participated in the present study, 1,138 of whom were submitted for an oral exam. The mean age was 39.55 ± 3.31 years (median: 39 years). The majority of the interviewees were women (65.5%), had some amount of schooling (98.0%) and were either married or in a stable union (67.5%). The monthly minimum salary at the time of the survey was R$510 (equivalent to US$300). The vast majority of participants reported a monthly household income ranging from one to three times the minimum salary (US$300 to US$900) (98.2%). The monthly household income ranged from US$0.00 to US$5,882.35, and the monthly income per capita ranged from US$0.00 to US$5,176.47.

**Figure 3:**
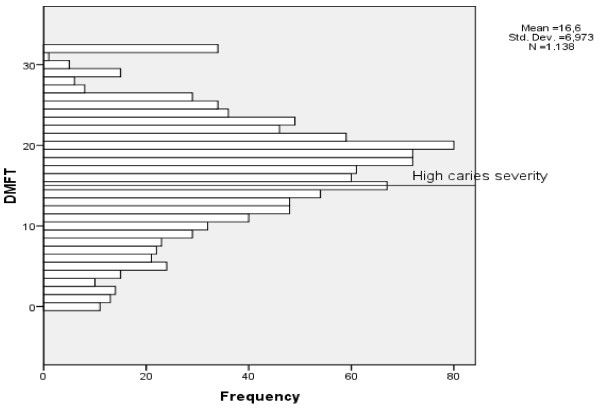
Distribution of individuals based on the severity of dental caries (DMFT ≥ 14 = high severity).

The majority of participants (68.5%) had high caries severity (Figure 3). Table 1 displays the distribution of the interviewees according to the independent variables. A total of 276 individuals reported participating in groups, 255 of whom specified the type of group (173 reported participating in religious groups, accounting for 67.84% of all those who participated in groups).

**Table 1:** Distribution of adults (N = 1150) according to individual characteristics, social structure and social context

**Variable**	**N**^**§**^	**%**	**Variable**	**N**^**§**^	**%**
*Gender*			Satisfaction with access to services *		
Female	753	65.5	Yes	442	39.1
Male	397	34.5	No	687	60.9
*Age*			Services used most *		
35-39 years	576	50.1	Others	783	70.2
40-44 years	574	49.9	Public	332	29.8
Marital status*			*Problem obtaining appointment*		
Married/Stable union	776	67.9	No	612	66.4
Others	367	32.1	Yes	309	33.6
Has negative feelings *			Household income*		
No	842	77.2	> R$1,020 (> US$600)	558	48.5
Yes	249	22.8	≤ R$1,020 (≤ US$600)	592	51.5
Considers self happy *			Schooling*		
Yes	843	74.7	University	95	8.3
No	286	25.3	Non-university	1,050	91.7
Capable of coping with stress*			*Participation in groups*		
Yes	1,011	90.2	Yes	276	24.0
No	110	9.8	No	874	76.0
Food preference *			Has persons willing to assist financially *		
Salty	822	81.3	Yes	692	60.6
Sweet	189	18.7	No	450	39.4
Use of dental floss *			Has security at home *		
Yes	796	72.8	Yes	391	34.6
No	297	27.2	No	738	65.4
Use of tooth paste *			Community has petitioned authorities for benefits *		
Yes	933	96.6	Yes	494	45.0
No	33	3.4	No	603	55.0
Regular visits to dentist *			*Capable of making decisions (empowerment)*		
Yes	399	35.9	Yes	845	74.5
No	712	64.1	No	289	25.5

*presence of missing data, adjusted percentage.

^§^N = number of adults examined.

In the bivariate analysis, the severity of dental caries was associated with the demographic profile, health-related behavior, psychological reactions, socioeconomic status, use of health services and social capital variables (Figure 1). Caries severity was significantly associated with gender (p = 0.049), age (p = 0.005), regular visits to the dentist (p = 0.004), the community having petitioned political leaders for neighborhood benefits (p = 0.004) and empowerment (p = 0.028) (Table 2).

**Table 2:** Bivariate and multivariate analyses, Poisson regression; adults aged 35 to 44 years (N = 1138)

**Variables**	**Severity of caries DMFT**		Prevalence ratio PR (95%CI)*	
**Demographic profile and health-related behavior (individual level)**	**DMFT <14 N ( %)**	**DMFT ≥ 14 N ( %)**	**Bivariate p**	**Multivariate p**
*Gender*				
Male	136 (35.3)	249 (67.7)	1	
Female	214 (28.4)	539 (71.6)	1.11 (1.00-1.24) p = 0.049	-
*Age*				
35-39 years	207 (36.4)	362 (63.6)	1	1
40-44 years	143 (25.1)	426 (74.9)	1.15 (1.04-1.26) p = 0.005	1.15 (1.04-1.26) ****p = 0.007
*Marital status*^§^				
Others	124 (34.3)	238 (65.7)	1	
Married/stable union	222 (28.9)	547 (71.1)	1.05 (0.94-1.17) p = 0.386	-
*Food preference*^§^				
Salty	252 (30.7)	570 (69.3)	1	
Sweet	55 (29.1)	134 (70.9)	1.02 (0.89-1.18) p = 0.746	-
*Use of dental floss*^§^				
No	91 (30.6)	206 (69.4)	1	
Yes	242 (30.4)	554 (69.6)	1.02 (0.92-1.14) p = 0.677	-
*Use of toothpaste*^§^				
No	10 (30.3)	23 (69.7)	1	
Yes	278 (29.8)	655 (70.2)	1.19 (0.74-1.90) p = 0.472	-
*Regular visits to dentist*^§^				
No	238 (33.4)	474 (66.6)	1	
Yes	103 (25.8)	296 (74.2)	1.15 (1.05-1.27) p = 0.004	1.18 (1.07-1.30) p = 0.001
**Psychological reactions (individual level)**	**DMFT <14 N (%)**	**DMFT ≥ 14 N (%)**	PR(95%CI)* Bivariate p	PR(95%CI)* Multivariate p
*Has negative feelings*^§^				
Yes	82 (32.9)	167 (67.1)	1	
No	249 (29.6)	593 (70.4)	1.08 (0.95-1.23) p = 0.234	-
*Considers self happy*^§^				
Yes	266 (31.6)	577 (68.4)	1	
No	80 (28.0)	206 (72.0)	1.00 (0.89-1.12) p = 0.995	-
*Capable of coping with stress*^§^				
No	36 (32.7)	74 (67.3)	1	
Yes	306 (30.3)	705 (69.7)	1.07 (0.89-1.29) p = 0.455	-
**Socioeconomic status and use of healthcare services (social structure)**	**DMFT < 14 N (%)**	**DMFT ≥ 14 N (%)**	PR(95%CI)* Bivariate p	PR(95%CI)* Multivariate p
*Satisfaction with access to services*				
Yes	138 (31.2)	304 (68.8)	1	
No	208 (30.3)	479 (69.7)	1.04 (0.94-1.15) p = 0.482	-
*Services most used*^§^				
Public	105 (31.6)	227 (68.4)	1	
Others	235 (30.0)	548 (70.0)	1.01 (0.91-1.12) p = 0.854	-
*Problem obtaining appointment*				
No	183 (29.9)	429 (70.1)	1	
Yes	89 (28.8)	220 (71.2)	1.02 (0.91-1.13) p = 0.759	-
*Monthly household income*^§^				
> US$600	191 (34.6)	361 (65.4)	1	1
≤ US$600	159 (27.1)	427 (72.9)	1.09 (0.99-1.20) p = 0.087	1.11 (1.01-1.23) p = 0.037
Schooling*				
University	34 (36.2)	60 (63.8)	1	
Non-university	316 (30.4)	723 (69.6) 1	1.17 (0.95-1.46) p = 0.147	-
**Social capital (social context)**	**DMFT <14 N ( %)**	**DMFT ≥ 14 N ( %)**	PR(95%CI)* Bivariate p	PR(95%CI)* Multivariate p
*Participation in groups*				
No	274 (31.6)	592 (68.4)	1	
Yes	76 (27.9)	196 (72.1)	1.08 (0.97-1.20) p = 0.143	-
*Has persons willing to assist financially*^§^				
Yes	216 (31.6)	468 (68.4)	1	
No	131 (29.4)	315 (70.6)	1.01 (0.92-1.12) p = 0.817	-
*Security at home*^§^				
Yes	135 (34.5)	256 (65.5)	1	
No	213 (28.9)	525 (71.1)	1.07 (0.96-1.18) p = 0.213	-
*Community petitioned authorities for neighborhood benefits*^§^				
Yes	165 (33.4)	329 (66.6)	1	1
No	175 (29.0)	428 (71.0)	1.16 (1.05-1.28) p = 0.004	1.16 (1.05-1.28) p = 0.003
*Capable of making decisions*				
Yes	273 (32.3)	572 (67.7)	1	1
No	75 (26.0)	214 (74.0)	1.12 (1.01-1.24) p = 0.028	1.12 (1.01-1.24) p = 0.012

*PR- prevalence ratio; CI- confidence interval; values adjusted for design effect.

^§^presence of missing data, adjusted percentage.

The multivariate analysis was performed with all variables that achieved a p-value of p ≤ 0.20 (gender, age, regular visits to the dentist, participation in groups, empowerment, monthly household income, schooling and petitioning leaders for neighborhood benefits). The caries severity remained significantly associated with age, regular visits to the dentist, communities having petitioned political leaders for neighborhood benefits, monthly household income and a feeling of empowerment. The prevalence of high caries severity among those aged 40 to 44 years was 1.15-fold (95%CI: 1.04 to 1.26) greater than among those aged 35 to 39 years. A greater prevalence of high caries severity was found among those who frequently visited the dentist (PR = 1.18; 95%CI: 1.07 to 1.30) in comparison to those who did not make regular visits to the dentist. A greater prevalence of high caries severity was also found among those with a lower income (PR = 1.11; 95%CI: 1.01 to 1.23), those who reported that their neighborhood did not come together in the previous year to petition political leaders for benefits in comparison to those who did (PR = 1.16; 95%CI: 1.05-1.28) and those who felt unable to make decisions (without empowerment) (PR = 1.12; 95%CI: 1.01 to 1.24) (Table 2).

## Discussion

Adults aged 35 to 44 years compose the index age group for the assessment of health [18]. Because the data collection in the present study was carried out at domiciles and involved adults in this age group, a greater level of participation by women was expected. According to the Brazilian Employment and Unemployment Survey [29] supported by the Labor Ministry and Workers’ Assistance Fund, the unemployment rate in metropolitan Belo Horizonte in 2007 was higher for women than men (15.9% and 8.9%, respectively), which may explain the greater number of women in the present study, as there was a greater chance of encountering women at home during the survey.

Considerable variation was found in household income, ranging from individuals with no income to those with a household income far above the national average (approximately 20 times the minimum salary). This finding demonstrates the considerable disparity in the distribution of income among the residents of metropolitan Belo Horizonte. Indeed, the entire country of Brazil displays differences in income between the richest and poorest inhabitants.

The majority of adults had high caries severity, demonstrating that this oral condition compromises a greater number of teeth among most adults. Based on our analysis of the independent variables in relation to the severity of dental caries, the majority of individuals had greater caries severity (DMFT ≥ 14) with respect to all of the variables studied. Moreover, age, regular visits to the dentist, monthly household income, petitions for neighborhood benefits and the capacity for decision making exerted a significant influence over the severity of caries. These variables comprise the individual level (demographic and behavioral), the social status of the individual (household income) and the social context perceived by the individual (social capital). Variables composing the psychological level (psychological reactions of the individual) did not remain associated with dental caries; thus, this level was the only one not to exhibit an association with the severity of dental caries.

The older age group (40 to 44 years) had a greater prevalence of high caries severity. This was expected, as the DMFT index also considers the past history of decayed, missing and filled teeth. Thus, the caries index is largely affected by the treatment history, which, to some extent, may explain the finding that greater caries severity was associated with regular visits to the dentist. Although multiple studies demonstrate that regular visits to the dentist constitute an important factor for reducing the severity of dental caries and are a recognized determinant of differences in the prevalence of caries between populations [3,30], the present study found contrary results. It is likely that the individuals in the present sample who most sought dental services were those with a greater number of dental caries. Another possible explanation is that dental practice is predominantly restorative and that more visits may signify overtreatment, as the emphasis given to oral health problems remains centered on traditional restorative treatment, which generates an increase in individuals with a large number of teeth having undergone some type of clinical intervention [31]. Moreover, the execution of restorative treatment is an isolated action that does not consider preventive efforts regarding oral conditions [32]. This latter explanation is more plausible considering that the contribution of filled component for the DMFT is higher among the group that visited dentist regularly comparing to the group that did not visit dentist [data not shown].

The association between a low monthly income and greater prevalence of high caries severity is in agreement with the findings of previous studies describing a worse oral health status among individuals with a low income [12,33,34]. A previous study concluded that individuals who are unable to afford private dental services had a 2.5-fold greater chance of having new carious lesions in comparison with those able to afford private treatment, even with difficulties (p <0.05) [35]. Moreover, not only is a lower household income associated with dental caries, but income inequities between cities and countries are also associated with this condition [36,37]. Women had high caries severity as compared to their male counterparts in the bivariate analysis. In the multivariate analysis, gender was not associated to dental caries. So, considering this result and that unemployment rate in the study area (metropolitan Belo Horizonte) was higher in female population than in the male [29], the high caries severity in female is probably a confounding.

The prevalence of high caries severity was 16% greater among those reporting not having petitioned political leaders for neighborhood benefits and 12% greater among those who felt unable to change the direction of their lives (lack of empowerment). Greater social capital may be associated with the placement of a greater value on health, thereby contributing to lesser caries severity. Social capital can be defined as the characteristics of the organization of a society, such as interpersonal trust, the norms of reciprocity and solidarity networks. The features of a social group can capacitate its participants with regard to more efficient collective actions in the quest for common objectives [38].

The capacity for decision making and petitioning members of the government represents the authority, empowerment and political action dimension of social capital, which refers to the broadening of the resources and capacities of individuals regarding negotiating with government institutions as well as holding these institutions accountable for the wellbeing of the community. As a dimension aimed at enhancing control over the decisions that affect the daily lives of individuals, this dimension is considered to transcend the concept of social capital [22]. In Brazil, the social control proposed by the public healthcare system ensures the participation of individuals in decision-making forums [39]. Such social control is directly related to the accumulation of social capital in Brazilian society [40]. Thus, community participation allows for greater influence over the definition of healthcare priorities.

The lesser prevalence of high caries severity among adults who had petitioned for neighborhood benefits and those who felt capable of changing the direction of their lives demonstrates that social context and empowerment regarding decision making can influence the health-illness process. These results underscore the importance of empowerment, which allows collective actions and active participation on the part of the community in social decision making and the quest for social rights. This may contribute to the reduced severity of dental caries, as a community without empowerment is more susceptible to health conditions. Another important contribution of the present study regards the need to unite the health sciences and social sciences for the implementation of interdisciplinary actions that can contribute to the prevention of dental caries in the population. Thus, there is a need for new paths in the field of collective health toward a more participative population, which could contribute to health promotion.

Furthermore, health is also influenced by an individual’s lifestyle and occupation as well as psychosocial, socio-demographic and dental care characteristics, and it is therefore a complex phenomenon. As such, measuring only part of this phenomenon constitutes an incomplete assessment [41]. Previous studies have demonstrated the influence of social capital [8,42] and social context [3,43] over oral health. However, a large number of studies have been conducted with methods that overlook the effects of social context. The understanding of the oral health status of the population in the present study provides a basis for the implementation of health programs that seek to empower individuals, capacitating them for decision making and proactive attitudes, such as the petitioning of political leaders and authorities for community benefits, as greater caries severity was associated with feelings of a lack of empowerment and the absence of community meetings to petition for neighborhood benefits.

However, beyond the relationship between oral health status and determinants of the individual and social context levels, the findings of the present study underscore the need for combined efforts among the different sectors of society regarding the understanding of the singularity and complexity of human beings. The interaction of diverse factors in the health-illness process requires a multiplicity of approaches and definitions, and the health sector alone is not capable of affecting changes in the actual health situation of population groups. Thus, there is a need to strengthen interdisciplinary actions with respect to individual and social rights to encourage changes in the oral health status of the population. The results also reinforce the need to implement health promotion programs aimed to strengthen social cohesion by empowering and training the population to decision making and community petitions. Another study recommended that oral health professionals must propose actions toward poverty reduction and contribute to improving relations with the needy members of society, develop strategies for positive and effective interactions as well as improving access to dental services for the poor. The fight against poverty has been justified on various grounds, including human rights and social justice [44].

The present investigation has limitations that should be addressed. The study was conducted with only part of the population of metropolitan Belo Horizonte, the state capital of Minas Gerais, Brazil. However, measures were taken to make the sample more representative of the region studied, such as the grouping of municipalities based on population size, the randomized selection of sampling units and the use of weight of expansion in the statistical analysis to compensate for unequal probabilities among the elements of the sample due to conglomerate sampling. Another limitation is the fact that the DMFT index is not sensitive for measuring the impact of the social aspects that influence oral health [45]. This index have others limitations. When we analyzed the F component it is believed that not all teeth were previously decayed. This index is subject also to the error of including other conditions or diseases not associated with dental caries [46]. However, it is the most frequently employed index worldwide. Due to the cross-sectional design, no cause-and-effect conclusions may be drawn regarding the associations between the severity of dental caries and the variables that remained in the final model. Moreover, the possibility of underestimating the total number of teeth with caries in the population studied should be considered, as the criterion for the diagnosis of dental caries was that proposed for epidemiological surveys by the World Health Organization, without the use of x-rays, which hinders the diagnosis of occlusal and inter-proximal caries. Moreover, other indicators of psychological conditions could be tested in other oral health surveys. Despite these limitations, the present study is important, as it is the first investigation to evaluate the oral health of adults in a metropolitan region of a major city in Brazil.

## Conclusions

The findings of the present study revealed high caries severity in adults aged 35 to 44 years, which was associated with individual characteristics, health-related behavior and factors related to social structure and context. These findings underscore the importance of considering the social determinants involved in the health-illness process when carrying out epidemiological studies on dental caries.

## Competing interests

The authors declare that there are no conflicts of interest related to the present study.

## Authors’ contributions

SMC was responsible for the acquisition of data, the analysis and interpretation of data and the organizing and drafting of the paper. MV was responsible for the analysis and interpretation of the data and contributed to the critical evaluation of the manuscript. JPAH was responsible for the analysis and interpretation of data. MHNGA was responsible for the study design, supervision and data collection as well as assisted with the analysis, the interpretation of the data and the writing of the paper. All authors have given final approval of the version to be published. All authors read and approved the final manuscript.

## Pre-publication history

The pre-publication history for this paper can be accessed here:

http://www.biomedcentral.com/1472-6831/12/25/prepub
